# P-857. Serratia Bacteremia: Risk Factors, Complications and Outcomes

**DOI:** 10.1093/ofid/ofae631.1049

**Published:** 2025-01-29

**Authors:** Roberta Monardo, Larry Park, Felicia Ruffin, Rachel E Korn, Parisa Farahani, Antonella Castagna, Marco Ripa, Vance G Fowler, Joshua T Thaden, Stacey Maskarinec

**Affiliations:** Vita-Salute San Raffaele University, Milan, Lombardia, Italy; Duke University Department of Medicine, Durham, North Carolina; Duke University Medical Center, Durham, NC; Duke University Medical Center, Durham, NC; Department of Medicine, Duke University, Roanoke, Virginia; IRCCS San Raffaele Hospital and Vita-Salute San Raffaele University, Milano, Lombardia, Italy; San Raffaele University Hospital, Milan, Lombardia, Italy; Duke University Medical Center, Durham, NC; Duke University School of Medicine, Durham, NC; Duke University Medical Center, Durham, NC

## Abstract

**Background:**

Previously considered a rare opportunistic pathogen, *Serratia* is now an emerging cause of bacteremia. However, the demographics and outcomes of patients with *Serratia* bacteremia remain poorly understood. We thus compared the risk factors and outcomes of patients with *Serratia* bacteremia relative to other *Enterobacterales* species in a prospectively ascertained cohort of hospitalized patients.

Rates of device infection in patients with medical devices.

**Figure:**
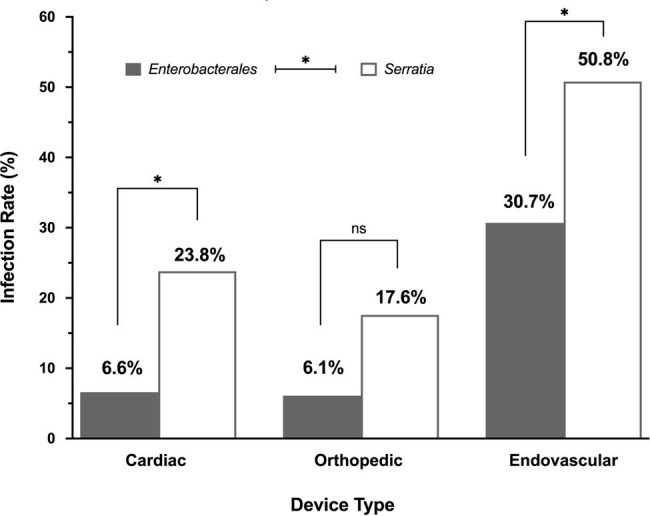

Rates of device infection in patients with medical devices stratified by device type. P values ≤0.05 are considered statistically significant and indicated (*).

**Methods:**

Patients with gram-negative bacteremia (GNB) were prospectively enrolled from 2002-2021 at Duke University. Clinical characteristics and outcomes were compared among patients with bacteremia due to *Serratia* and other *Enterobacterales*. Multivariable logistic regression models were used to determine features independently associated with outcomes.

Risk factors associated with device-associated infection.

**Figure:**
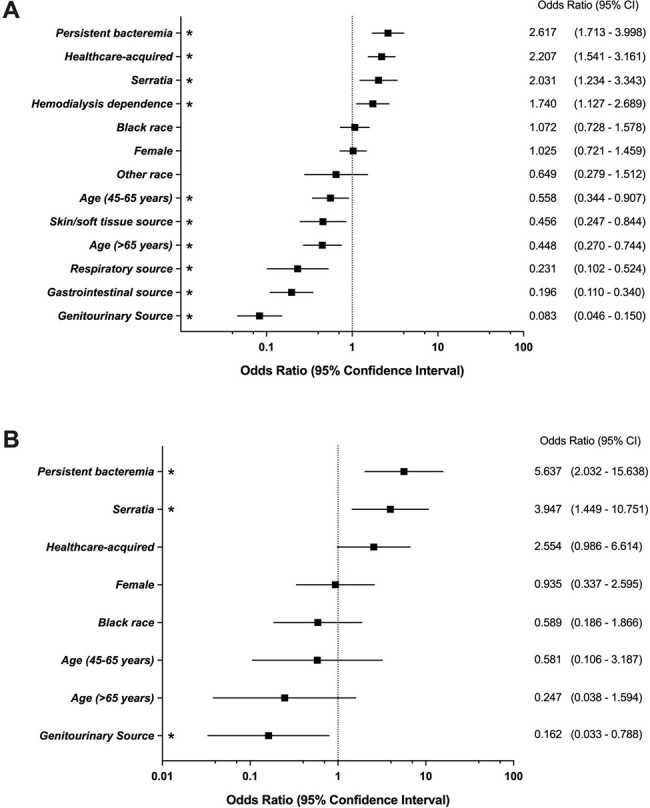

Risk factors associated with device-associated infection. The multivariable models of factors associated with underlying device infection in patients with any medical device (A) and only cardiac devices (B) and gram-negative bacteremia are shown. P values ≤0.05 are considered statistically significant and indicated (*).

**Results:**

Among 2676 patients with GNB, 173 (6.5%) had *Serratia* bacteremia. Patients with *Serratia* bacteremia, relative to GNB caused by other *Enterobacterales*, were more likely to have a medical device (59% vs 35.6%; *P*< 0.001), device-associated infection (42.2% vs. 21.3%, *P*< 0.001), and persistent bacteremia (23.2% vs. 12.9%; *P*< 0.001). Among all patients with a medical device (n = 993; 37.1%), *Serratia* was associated with an increased risk of any device infection in an adjusted model (odds ratio (OR) 2.031; 95% confidence interval (CI), 1.234-3.343). The risk for device infection was highest among patients with cardiac devices (OR 3.947, 95% CI 1.449-10.751).

**Conclusion:**

Compared to patients with GNB due to other *Enterobacterales*, patients with *Serratia* bacteremia were more likely to have a device infection in general, a cardiac device-associated infection in particular, and persistent bacteremia.

**Disclosures:**

**Antonella Castagna, MD**, Bristol-Myers Squibb: Advisor/Consultant|Gilead Sciences, Inc.: Advisor/Consultant|Gilead Sciences, Inc.: Grant/Research Support|Gilead Sciences, Inc.: Honoraria|Janssen: Advisor/Consultant|Janssen: Grant/Research Support|Janssen: Honoraria|Merck Sharp & Dohme: Advisor/Consultant|Merck Sharp & Dohme: Grant/Research Support|Merck Sharp & Dohme: Honoraria|ViiV Healthcare: Advisor/Consultant|ViiV Healthcare: Grant/Research Support|ViiV Healthcare: Honoraria **Vance G. Fowler, MD, MHS**, Affinergy: Advisor/Consultant|ArcBio: Stocks/Bonds (Private Company)|Armata: Advisor/Consultant|Astra Zeneca: Advisor/Consultant|Astra Zeneca: Grant/Research Support|Basilea: Advisor/Consultant|Basilea: Grant/Research Support|ContraFect: Advisor/Consultant|ContraFect: Grant/Research Support|Debiopharm: Advisor/Consultant|Destiny: Advisor/Consultant|EDE: Grant/Research Support|Genentech: Advisor/Consultant|Genentech: Grant/Research Support|GSK: Advisor/Consultant|Janssen: Advisor/Consultant|Karius: Grant/Research Support|MedImmune: Grant/Research Support|Merck: Grant/Research Support|sepsis diagnostics: Patent pending|UptoDate: Royalties|Valanbuio: Stocks/Bonds (Private Company)|Valanbuio: Stocks/Bonds (Private Company) **Joshua T. Thaden, MD, PhD**, National Institutes of Health K08 AI171183 (Thaden): Grant/Research Support **Stacey Maskarinec, MD, PHD**, National Institutes of Health K23 HL159275 (Maskarinec): Grant/Research Support

